# A 5-Year Audit of Accidental Dural Punctures, Postdural Puncture Headaches, and Failed Regional Anesthetics at a Tertiary-Care Medical Center

**DOI:** 10.1100/tsw.2009.94

**Published:** 2009-08-01

**Authors:** Sukhdip Singh, Shagufta Y. Chaudry, Amy L. Phelps, Manuel C Vallejo

**Affiliations:** ^1^ Magee-Womens Hospital, Department of Anesthesiology, University of Pittsburgh School of Medicine, USA; ^2^ Duquesne University, School of Business, Pittsburgh, PA, USA

**Keywords:** accidental dural puncture, postdural puncture headache, failed regional, maternal outcome

## Abstract

Obstetric anesthesia-related complications occur as a result of labor epidural or spinal placement. The purpose of this continuous quality-improvement audit was to review the occurrence of accidental dural punctures (ADPs), postdural puncture headaches (PDPHs), and failed regional anesthetics at an academic tertiary-care medical center over a 5-year period. Obstetric anesthesia complications contained in three databases consisting of ADPs, PDPHs, and failed regional anesthetics were matched to a perinatal database, with no complications serving as controls. Of the 40,894 consecutive parturients, there were 765 documented complications. Complication rates were 0.73% (95% CI: 0.65–0.82) for ADP, 0.49% (95% CI: 0.43–0.56) for PDPH, and 0.65% (95% CI: 0.57–0.73) for failed regional anesthetic. When compared to the no complication group, factors associated with obstetric anesthesia complications included increased weight and BMI (*p* < 0.01), epidural block (*p* < 0.01), and vaginal delivery (*p*< 0.01).

### INTRODUCTION

Accidental dural punctures (ADPs), postdural puncture headaches (PDPHs), and failed regional anesthetics occur during the course of epidural or spinal needle placement. ADP is a common complication of epidural insertion for labor analgesia, with a reported incidence of 1–5%[1,2,3,4,5,6,7,8]. Resultant PDPH following ADP can be very distressing and extremely disabling[1,7]. A failed regional anesthetic is also problematic because the parturient has inadequate analgesia, and requires another neuraxial block or a potentially emergent general anesthetic with its inherent complications. The purpose of this audit was to review the incidence of ADP, PDPH, and failed regional anesthetics at a tertiary-care, academic, teaching medical center over a continuous 5-year period and to determine contributing factors related to these complications.

### MATERIALS AND METHODS

This study received local Investigational Review Board approval. Magee-Womens Hospital is a tertiary-care, academic, teaching medical center with greater than 9,500 deliveries per year and an 89% regional anesthetic placement rate. Obstetric anesthesia complications contained in three databases consisting of ADPs, PDPHs, and failed regional anesthetics were matched to a perinatal database over a 5-year period, with no complications serving as controls.

For the purpose of quality assurance, the anesthesiology department maintains a database of all patients who develop complications, including ADPs, PDPHs and failed regional anesthetics. An ADP is defined as an inadvertent dural puncture by the epidural needle or catheter with return of cerebral spinal fluid (CSF), with or without aspiration, or a resultant dense subarachnoid block after a test dose of 3 ml of 1.5% lidocaine with 1:200,000 epinephrine. A PDPH was defined as the diagnosis of a PDPH requiring conservative treatment or a therapeutic blood patch. Criteria used to diagnosis a PDPH included a headache that was postural in nature (worse assuming an upright position), related in time to placement of regional anesthesia (epidural or spinal) for delivery, and associated with other characteristic PDPH symptoms, including nausea, neck pain, tinnitus, and photophobia. A failed regional anesthetic was defined as any neuraxial anesthetic (spinal or epidural) that was unsuccessful, requiring an additional anesthetic procedure (reinsertion). Resultant complications from an epidural block consisted of ADP, PDPH, and failed regional anesthetics. Resultant complications from a spinal block consisted of PDPH and a failed regional anesthetic (ADP was not counted as a complication for a spinal block) (Table 1). A parturient with an ADP and/or PDPH was counted as having only one epidural complication. Inclusion criteria included all parturients who were admitted and delivered vaginally and by cesarean section, with or without a regional anesthetic, at Magee-Womens Hospital from 2002 through 2006.

**TABLE 1**
**Demographic and Block Type Data**

| | No Complication Group ($n$ = 40,129) | Complication Group ($n$ = 765) | $p$ |
|---|---|---|---|
| Age (years) | 29.5 ± 6.2 | 29.6 ± 6.3 | 0.57 |
| Ht (cm) | 164.3 ± 6.9 | 164.6 ± 7.4 | 0.41 |
| Wt (lb) | 182.9 ± 37.6 | 189.7 ± 42.0 | <0.01 |
| BMI (kg/m$^2$) | 30.81 ± 5.92 | 31.80 ± 6.54 | <0.01 |
| Gravid | 2 (1–25) | 2 (1–10) | 0.45 |
| Parity | 1 (0–14) | 1 (0–8) | 0.33 |
| Race | | | 0.23 |
| Caucasian (%) | 30,309 (75.5%) | 559 (73.1%) | — |
| African American (%) | 7,545 (18.8%) | 154 (20.1%) | — |
| Other race (%) | 2,275 (5.7%) | 52 (6.8%) | — |
| Type of block | | | <0.01 |
| Epidural block (%) | 29,856 (74.4%) | 626 (81.8%) | <0.01 |
| Spinal block (%) | 5,939 (14.8%) | 100 (13.1%) | 0.16 |
| Other (%) | 4,334 (10.8%) | 39 (5.1%) | <0.01 |
| Vag Del (%) | 30,097 (75%) | 536 (70%) | <0.01 |
| C/S Del (%) | 10,032 (25%) | 229 (30%) | <0.01 |

Legend: Data are mean ± SD; median with range in parenthesis or percentages in parenthesis; BMI = body mass index; Vag Del = vaginal delivery; C/S = cesarean section; Other = no spinal or epidural anesthetic intervention.

In order to identify all patients who developed an anesthesia-related complication as a result of epidural or spinal placement, patients were identified from three data sources. The first data source came from the Magee-Womens Hospital Obstetrical Tracevue anesthesia electronic record, which is used on all patients who are admitted and receive a regional anesthetic in the labor and delivery suite. A sample of the anesthesia electronic record appears in the Appendix. The electronic record was queried for anesthetic-related complications, which included ADP, PDPH, and failed regional anesthetic. The second data source came from the medical records identified as having a regional anesthetic-related complication. The third data source came from a postcomplication anesthesia form, which is a written record used by the anesthesia department to follow patients who have had a regional anesthetic complication throughout their hospital stay. For quality assurance purposes, our department employs a dedicated nurse practitioner who follows up and collects data on all obstetric anesthesia-related complications.

In order to provide a denominator for calculation of the complication rates, parturients who had an anesthesia-related complication were matched to the Magee-Womens Hospital Obstetric and Medical Information (MOMI) perinatal database that includes all vaginal deliveries, cesarean sections, and spinal and epidural insertions at our institution. Parturients in the perinatal MOMI database who did not have an anesthesia-related complication served as controls for comparison.

### Statistical Analysis

Interval data are presented as mean ± SD and analyzed using the student *t*-test[9]. Ordinal data are presented as median with range in parentheses. Comparisons between the two groups were analyzed using the Mann-Whitney nonparametric test. Nominal data are presented as a frequency with a percent in parentheses, and analyzed using the Chi-square or Fisher's exact test where appropriate; $p < 0.05$ is considered statistically significant. When a significant change occurred in a complication rate over the 5-year period, a simple Bonferroni approach was used, where a $p < 0.005$ was considered significant for each of the ten comparison tests between the 5 years.

### RESULTS

Demographic data comparing the complication group to the no complication group are presented in Table 1. No differences were noted with respect to demographic data except that the complication group was heavier than the no complication group. Additionally, there were no differences with respect to racial distribution comparing the no complication to the complication group.

From January 3, 2002 to December 30, 2006, a total of 765 documented complications consisting of ADPs, PDPHs, and failed regional anesthetics were identified and matched to the perinatal MOMI database that had 40,894 deliveries (7,728 in 2002; 7,788 in 2003; 8,034 in 2004; 8,603 in 2005; and 8,741 in 2006) over this time period.

Among the 765 complications, 39.2% (n = 300) were ADPs, 26.3% (n = 201) were PDPHs, and 34.5% (n = 264) were failed regional anesthetics. There was no difference in the ADP rates over the 5-year period, $p = 0.61$ (Table 2). However, there was a significant increase in the PDPH rate in 2004 and 2005 compared to 2002 and 2003, $p < 0.005$ (Table 2). Likewise, there was a significant increase in the failed regional rate in 2003 compared to 2002 ($p < 0.005$); in 2005 compared to 2003 and 2004 ($p < 0.005$); and in 2006 compared to 2002, 2003, 2004, and 2005 ($p < 0.005$) (Table 2).

Chi-square analysis indicated that the type of anesthetic block (epidural or spinal) was significantly related to complication rate ($p < 0.001$). More epidurals were performed in the complication group as compared to the no complication group (81.8% compared to 74.4%, $p = 0.001$), while no significant difference was found in the rate of spinals between groups (13.1% compared to 14.80%, $p = 0.378$).

**TABLE 2**
**Obstetric Anesthesia-Related Complication Rates per Year**

| | 2002 | 2003 | 2004 | 2005 | 2006 | *p* |
|---|---|---|---|---|---|---|
| ADP | 0.76% | 0.79% | 0.62% | 0.67% | 0.79% | 0.607 |
| PDPH | 0.34% | 0.20% | 0.70%* # | 0.72%* # | 0.53% | <0.001 |
| Failed regional | 0.54% | 0.17%* | 0.27% | 0.61%# @ | 1.53%* # @ ! | <0.001 |

Legend: * = $p < 0.005$ compared to 2002, # = $p < 0.005$ compared to 2003, @ = $p < 0.005$ compared to 2004, ! = p < 0.005 compared to 2005.

Table 3 presents overall group provider complication rates between anesthesia staff and resident trainees. At Magee-Womens Hospital, there are 24 staff obstetric anesthesiologists. Fifty-two percent (52%) of anesthesia residents are second-year (CA2) residents, 31% are third-year (CA3) residents, and 17% are first-year (CA1) residents.

**TABLE 3**
**Percent Individual Group Provider Complication Rate**

| | Resident | Anesthesiologist | *p* |
|---|---|---|---|
| ADP rate | 1.65% | 0.92% | <0.01 |
| CA1 | 0.33% | — | — |
| CA2 | 0.87%* | — | — |
| CA3 | 0.45% | — | — |
| PDPH rate | 0.81% | 0.91% | 0.50 |
| Failed regional rate | 1.01% | 1.04% | 0.87 |

Legend: % = rate of individual group (resident trainee or staff anesthesiologist) complications; * = $p < 0.01$ compared to CA1 and CA3 residents; resident failure rates are not reported for the PDPH rate and failed regional rate as differences between groups were not significant.

The ADP rate was highest among the residents ($p < 0.01$) (Table 3). The ADP rate was also highest in the second year of training (CA2) compared to the first (CA1) and third year (CA3) of anesthesia residency ($p < 0.01$). No differences were noted with respect to resident trainee or resident year of training in the PDPH and failed regional rates compared to anesthesia staff (Table 3).

The vast majority of obstetric anesthesia-related complications consisted of ADPs, PDPHs, and failed regional anesthetics. The only other related complications during this time period consisted of residual back pain (n = 39), numbness and tingling in the lower extremities (n = 17), high spinal block requiring respiratory support (n = 6), and one epidural catheter that broke off during removal (n = 1), which was later removed under fluoroscopy. There were no cases of epidural hematoma formation, meningitis, infection, or permanent neurological injury.

### DISCUSSION

ADP or PDPH and failed regional anesthetics occur during the course of epidural or spinal anesthetic placement. Quality assurance data regarding obstetrical analgesia failure and complications are important because they can provide an understanding of failure trends, improve patient safety and outcomes, and ultimately lead to better patient care[6]. Quality assurance data can also shape future prospective studies designed to improve patient safety and outcomes.

When compared to the no complication group, factors associated with an obstetric anesthesia-related complication included epidural block ($p < 0.01$), vaginal delivery ($p < 0.01$), weight ($p < 0.01$), and BMI ($p < 0.01$). The significance between epidural insertion and vaginal delivery is related to the fact that patients who labor and deliver vaginally are most likely to receive an epidural at our institution. With epidural placement, the diameter of the epidural needle (17 gauge) is much larger than the diameter of the spinal needle (24 or 25 gauge), which is a known risk factor for PDPH[5]. Additionally, compared to the lean parturient, it is well known that obesity is a risk factor for ADP and initial failed labor epidural with subsequent epidural replacement because of the increased adipose tissue that makes identification of appropriate landmarks difficult and is also a factor in epidural catheter migration[10].

The incidence of ADP during the initiation of epidural analgesia/anesthesia in the obstetric population is reported in the literature to be between 0.04 and 6%[3,7]. The ADP rate at our institution has remained stable from 2002 through 2006 with a mean rate of 0.73% (Table 2). Choi et al. performed a meta-analysis of obstetrical studies and found that parturients have a 1.5% (1 in 67) risk of ADP with epidural insertion[4], and approximately half will result in a PDPH[4,5]. We found this same trend between ADP (0.73%) and PDPH (0.49%) in our study (Table 2).

Looking at the overall 5-year trends, there was a significant increase in PDPH and failed regional rates over the 5-year time period (Table 2). Possible reasons for our increased PDPH rate and failed regional rate include: (1) better electronic complication documentation and (2) an increase in overall number of residents trained over the 5-year period where a higher complication rate can be expected compared to nontraining institutions.

Compared to staff anesthesiologists, the ADP rate was highest among the residents (Table 3). This is most likely related to the fact that residents are not as experienced as staff anesthesiologists. Even advanced trainees in their second and third year of training may have an extended period of time between obstetric rotations (up to 1 year). As a result of our trainees having a higher rate of regional anesthesia complications, we have instituted an obstetrical regional anesthesia workshop, which is given to all new anesthesia residents in their first year of anesthesia training. This workshop consists of three parts. Part 1 is a web-based instructional video module component that describes the epidural and spinal kits used at our institution, proper patient positioning, labor epidural and spinal insertion techniques, and the administration of labor analgesia through continuous and patient-controlled epidural pumps. Part 2 is a web-based quiz with instructional feedback to assess learning comprehension of the modules. Part 3 is the hands-on learning component, where residents practice spinal and epidural insertion techniques on training mannequins with the guidance of teaching staff anesthesiologists.

The limitations of any retrospective study include the introduction of selection and reporting bias during data collection[6]. In order to limit this bias, we included several data sources and matched the cases to the perinatal MOMI database. Because the MOMI database is an obstetrical database, information regarding specific anesthesia-related information, number of needle attempts, skin site placements, number of epidural/spinals used for cesarean section and labor analgesia, number of combined spinal epidurals that produced an ADP, and timing of headaches relative to delivery was not available for analysis.

In conclusion, regional anesthetic complications are associated with increased weight and BMI, epidural placement, and vaginal delivery. In addition, we have presented the complication rates found at a teaching, tertiary-care, obstetric, academic medical center using the audited information contained in three large databases, and the implementation of an obstetrical regional anesthesia didactic and training simulation workshop given to all new anesthesia residents in their first year of anesthesia training for prevention of these complications.

## References

1. Baraz, R. and Collis, R.E. (2005) The management of accidental dural puncture during labour epidural analgesia: a survey of UK practice. *Anesthesia* **60**, 673–679.
2. Berger, C.W., Crosby, E.T., and Grodecki, W. (1998) North American survey of the management of dural puncture occurring during labour epidural analgesia. *Can. J. Anaesth.* **45**, 110–114.
3. Bucklin, B.A., Tinker, J.H., and Smith, C.V. (1999) Clinical dilemma: a patient with postdural puncture headache and acute leukemia. *Anesth. Analg*. **88**, 166–167.
4. Choi, P.T., Galinski, S.E., Takeuchi, L., Lucas, S., Tamayo, C., and Jadad, A.R. (2003) PDPH is a common complication of neuraxial blockade in parturients: a meta-analysis of obstetrical studies. *Can. J. Anaesth.* **50**, 460–469.
5. Gaiser, R. (2006) Postdural puncture headache. *Curr. Opin. Anaesthesiol.* **19**, 249–253.
6. Pan, P.H., Bogard, T.D., and Owen, M.D. (2004) Incidence and characteristics of failure in obstetrics neuraxial analgesia and anesthesia: a retrospective analysis of 19,259 deliveries. *Int. J. Obstet. Anesth*. **13**, 227–233.
7. Scavone, B.M., Wong, C.A., Sullivan, J.T., Yaghmour, E., Sherwani, S.S., and McCarthy, R.J. (2004) Efficacy of a prophylactic epidural blood patch in preventing post dural puncture headache in parturients after inadvertent dural puncture. *Anesthesiology* **101**, 1422–1427.
8. Vallejo, M.C., Mandell, G.L., Sabo, D.P., and Ramanathan, S. (2000) Postdural puncture headache: a randomized comparison of five spinal needles in obstetric patients. *Anesth. Analg*. **91**, 916–920.
9. Glanz, S.A. (1977) *Primer of Biostatistics*. 4th ed. McGraw-Hill, New York.
10. Vallejo, M.C. (2007) Anesthetic management of the morbidly obese parturient. *Curr. Opin. Anaesthesiol.* **20**, 175–180.

## APPENDIX

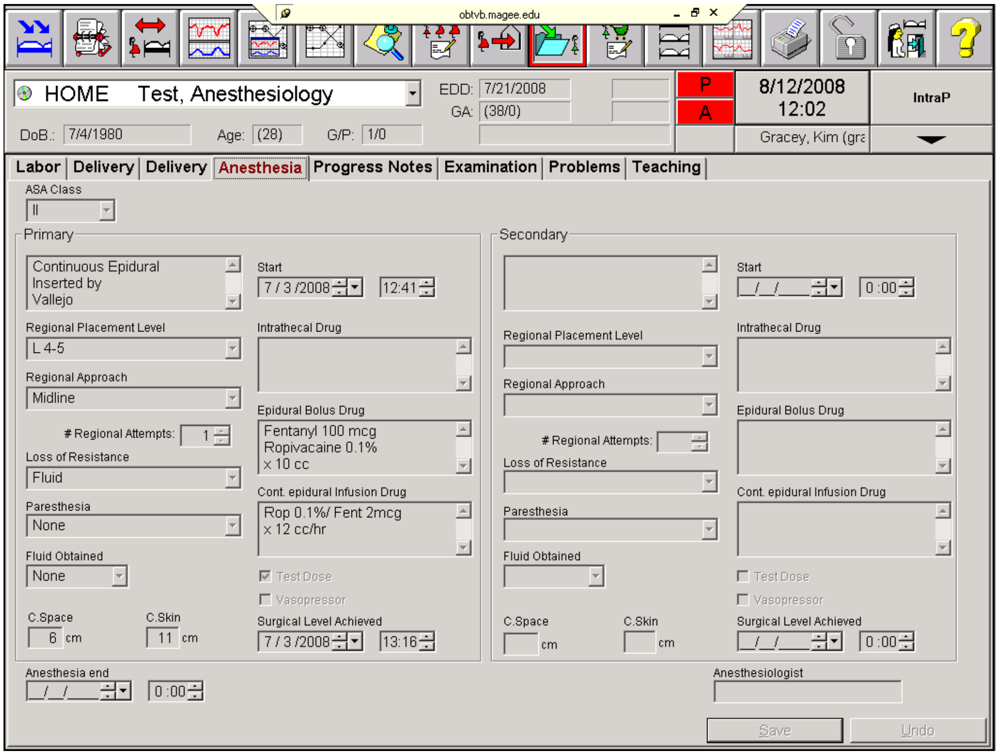

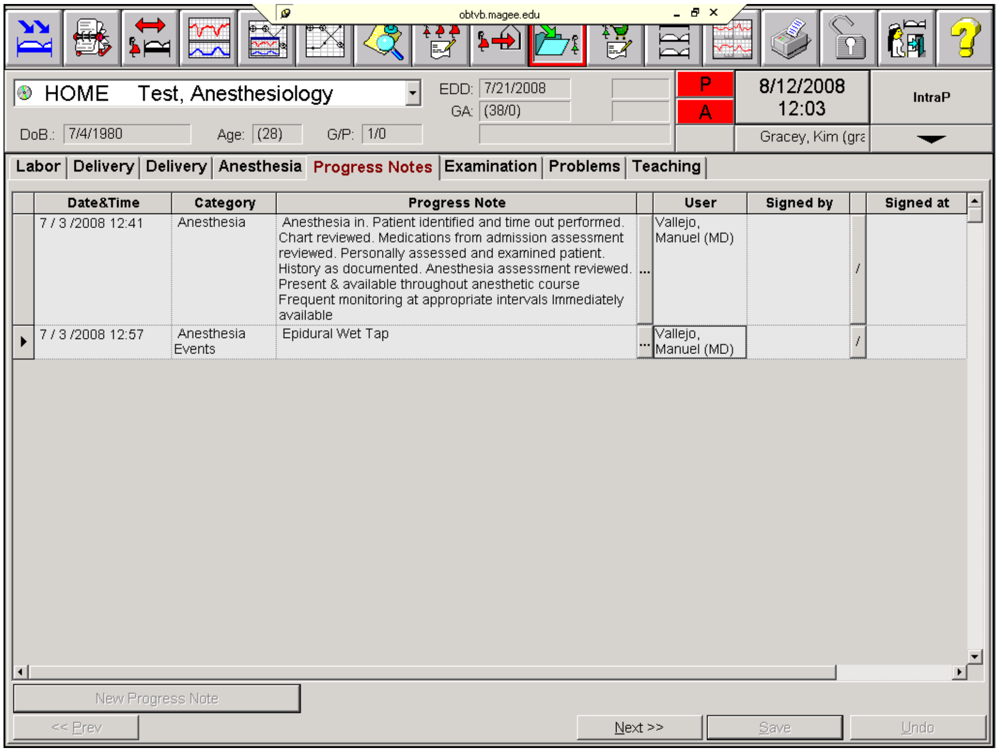

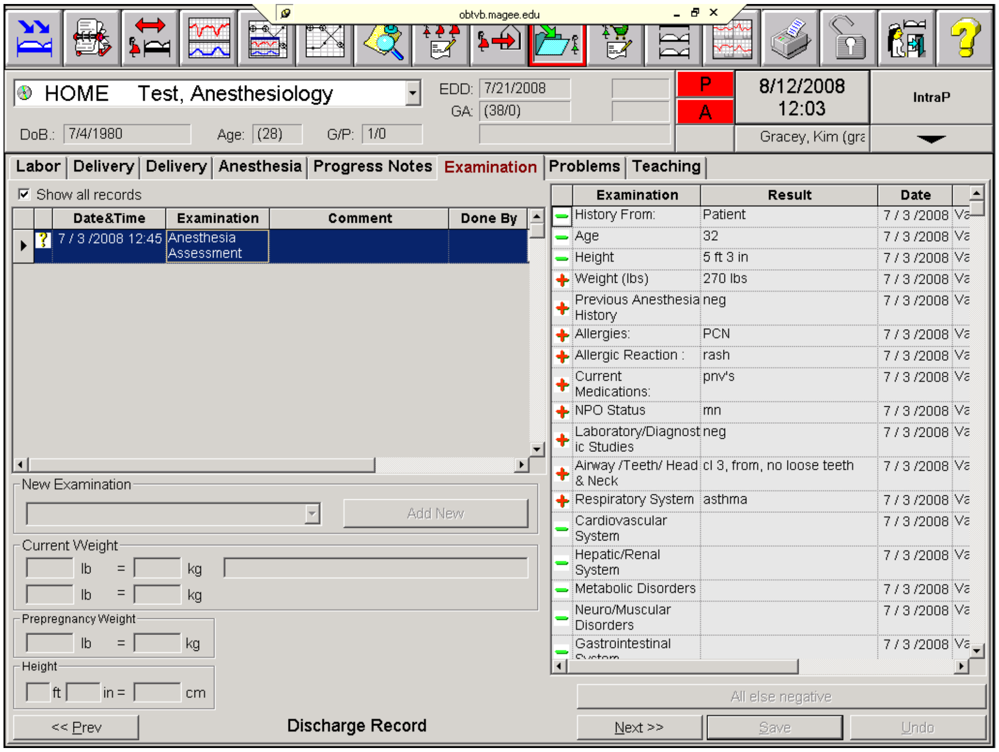

### Example of Anesthesia Electronic Medical Record obtvb.magee.edu
HOME Test, Anesthesiology
EDD: 7/21/2008
GA: (38/0)
DoB.: 7/4/1980
Age: (28)
G/P: 1/0
P
A
8/12/2008
12:03
IntraP
Gracey, Kim (gra
Labor
Delivery
Delivery
Anesthesia
Progress Notes
Examination
Problems
Teaching
Show all records
Date&Time
Examination
Comment
Done By
7 / 3 /2008 12:45
Anesthesia Assessment
New Examination
Add New
Current Weight
lb
kg
Prepregnancy Weight
Height
ft
in =
cm
Examination
Result
Date
History From:
Patient
Age
32
Height
5 ft 3 in
Weight (lbs)
270 lbs
Previous Anesthesia History
neg
Allergies:
PCN
Allergic Reaction :
rash
Current Medications:
pnv's
NPO Status
mn
Laboratory/Diagnostic Studies
neg
Airway /Teeth/ Head & Neck
cl 3, from, no loose teeth
Respiratory System
asthma
Cardiovascular System
Hepatic/Renal System
Metabolic Disorders
Neuro/Muscular Disorders
Gastrointestinal
7 / 3 /2008
All else negative
<< Prev
Discharge Record
Next >>
Save
Undo
